# Differential expression profile of DREB2 subfamily transcription factor genes in the dynamics of salt stress and post-stress recovery in tomato plants

**DOI:** 10.18699/vjgb-25-128

**Published:** 2025-12

**Authors:** M.A. Filyushin, A.V. Shchennikova, E.Z. Kochieva

**Affiliations:** Institute of Bioengineering, Research Center of Biotechnology of the Russian Academy of Sciences, Moscow, Russia; Institute of Bioengineering, Research Center of Biotechnology of the Russian Academy of Sciences, Moscow, Russia; Institute of Bioengineering, Research Center of Biotechnology of the Russian Academy of Sciences, Moscow, Russia

**Keywords:** tomato, Solanum lycopersicum L., salt stress, stress memory, memory phase, SlDREB2 transcription factors, gene expression, potential stress memory genes, томат, Solanum lycopersicum L., солевой стресс, стрессовая память, фаза памяти, транскрипционные факторы SlDREB2, экспрессия генов, гены-кандидаты стрессовой памяти

## Abstract

In response to stress, epigenetic modifications occur in the plant genome, which together form a stress memory that can be inherited and increases the efficiency of the plant's defense response to repeated stress events. Genes whose expression becomes the target of epigenetic modifications serve as biomarkers of stress memory. Their characteristic features are considered to be an expression profile that differs between responses to primary and repeated stress events, as well as long-term retention of changes after the stress is canceled. Tomato (Solanum lycopersicum L.) is an important vegetable crop whose yield decreases with soil salinity. Genes induced by salt stress include genes encoding transcription factors of the DREB2 (DEHYDRATION-RESPONSIVE ELEMENT-BINDING PROTEIN 2) subfamily. In this work, we evaluated the SlDREB2 genes of tomato as possible marker genes of salt stress memory. The expression of the genes SlDREB16, 20, 22, 24, 43, 44 and 46 was determined in the leaves of two plant varieties (Gnom, Otradnyi) with different degrees of salt tolerance in response to 24 h of NaCl exposure and in the dynamics of a long-term (14 days) post-stress recovery period. Significant genotype-specific fluctuations in the levels of gene transcripts were revealed both in the control and in the stressed plants. It was shown that during the long-term memory phase, gene expression returns to the control values either temporarily (SlDREB24, 44 and 46 in the moderately resistant Gnom variety after 7 days; after 14 days, the expression changed again) or slowly (SlDREB16 and 43 in the highly resistant Otradnyi variety after 14 days of recovery). Only two genes (SlDREB22 and 46) showed a similar between varieties pattern of expression fluctuations in the dynamics of stress and recovery, and the SlDREB20 gene was not expressed in either the control or the experiment. The data obtained suggest that the SlDREB2 subfamily genes (except SlDREB20) are involved in the response of S. lycopersicum to salt stress in a genotype-specific manner and can serve as markers of stress memory linked to the epigenetic regulation of tomato adaptation to salt stress. The SlDREB16, 28, 43 and 44 genes may contribute to the determination of differences in the mechanism of regulation of plant response to salt stress between salt-tolerant genotypes of S. lycopersicum. The obtained results can form the basis for further studies of the role of SlDREB2 genes in the epigenetic regulation of tomato plant adaptation to salt stress, which can be used in breeding salt-tolerant varieties

## Abstract (ru)

В ответ на стрессовое воздействие в геноме растения происходят эпигенетические модификации, вместе формирующие стрессовую память, которая может наследоваться и повышает эффективность защитной реакции растения на повторные стрессовые события. Гены, чья экспрессия становится мишенью эпигенетических модификаций, служат биомаркерами стрессовой памяти. Их характерными признаками считаются профиль экспрессии, различающийся между ответами на первичное и повторное стрессовые события, а также длительное удерживание изменений после отмены стресса. Томат (Solanum lycopersicum L.) – важная овощная культура, урожайность которой снижается при засолении почв. К генам, индуцируемым солевым стрессом, относятся гены транскрипционных факторов подсемейства DREB2 (DEHYDRATION-RESPONSIVE ELEMENT-BINDING PROTEIN 2). В настоящей работе проведена оценка генов SlDREB2 томата как возможных генов-маркеров памяти о солевом стрессе. Для этого в листьях растений двух сортов, Гном и Отрадный, с разной степенью солеустойчивости была определена экспрессия генов SlDREB16, 20, 22, 24, 43, 44 и 46 в ответ на 24-ч воздействие NaCl и в динамике продолжительного (14 дней) послестрессового периода восстановления. Выявлены значительные генотип- специфичные колебания уровней транскриптов генов как в контроле, так и в подвергнутых стрессу растениях. Показано, что в процессе длительной фазы памяти экспрессия генов возвращается к контрольным показателям либо временно (SlDREB24, 44 и 46 у среднеустойчивого сорта Гном через 7 дней; через 14 дней экспрессия снова меняется), либо медленно (SlDREB16 и 43 у высокоустойчивого сорта Отрадный через 14 дней восстановления). Было определено, что только два гена, SlDREB22 и 46, имеют сходный между сортами паттерн колебаний экспрессии в динамике стресса и восстановления, а ген SlDREB20 не экспрессируется ни в контроле, ни в опыте. Полученные данные позволили предположить, что гены подсемейства SlDREB2, кроме SlDREB20, участвуют в ответе S. lycopersicum на солевой стресс генотип-специфичным образом и могут служить маркерами стрессовой памяти, сцепленными с эпигенетической регуляцией адаптации томата к солевому стрессу. Гены SlDREB16, 28, 43 и 44 могут вносить вклад в определение различий в механизме регуляции ответа растений на солевой стресс между солеустойчивыми генотипами S. lycopersicum. Полученные результаты могут стать основой для дальнейших исследований роли генов SlDREB2 в эпигенетической регуляции адаптации растений томата к солевому стрессу, что может быть использовано в селекции солеустойчивых сортов.

## Introduction

The plant phenotype is formed through the combined action of the genotype and the epigenome, where the latter determines the plasticity of the phenotype depending on environmental conditions, including in response to various stress factors, which are often recurrent (Villagómez-Aranda et al., 2022). The initial (during the plant’s life cycle) experience of stress (priming) induces changes in the epigenome (DNA methylation, post-translational histone modifications, non-coding RNA activity, etc.), which enable a more effective response to repeated stress (stimulus) (Villagómez-Aranda et al., 2022).

The set of epigenetic marks that emerge during priming is called the plant’s stress memory, which can persist throughout the organism’s life cycle and be inherited (Villagómez-Aranda et al., 2022; Zuo et al., 2023). That is, the plant’s stress memory is the initial experience of effectively regulating the stress response, imprinted in the epigenome, which, upon a repeated stress event, can quickly trigger the transcriptomic and metabolomic changes necessary for protection (Villagómez-Aranda et al., 2022; Zuo et al., 2023).

Biomarkers of stress memory are generally considered to be individual genes (metabolites), the expression (metabolism) of which becomes the target of epigenetic modifications after priming (Aina et al., 2024). There may be many such markers. For example, drought stress memory in the model species Arabidopsis thaliana L. is associated with more than 2,000 genes (Ding et al., 2013). A comparison of this list with a similar set in Zea mays L. reduced the list to 556 genes as possible interspecific markers of plant memory about drought (Ding et al., 2014; Virlouvet et al., 2018; Jacques et al., 2021). When selecting candidate memory markers, the principle is that the level and/or direction of changes in gene expression (metabolite content) differs between responses to priming and stimulus, while genes (metabolites) not associated with memory respond equally to priming and stimulus (Friedrich et al., 2019; Bäurle, Trindade, 2020; Jacques et al., 2021). Another important criterion is that during the period between stress repeats (recovery, or memory phase), the expression of marker genes (metabolite content) is maintained at an altered level for a long time, while the expression of genes (metabolite content) not associated with memory quickly returns to control values (Friedrich et al., 2019; Bäurle, Trindade, 2020; Jacques et al., 2021).

An example of the criteria use is a metabolomic analysis of the halophyte Limonium angustebracteatum’s response to repeated drought and salt stresses, which identified various organic osmolytes and antioxidant compounds (including flavonoids) as potential markers of stress memory (Calone et al., 2023). Transcriptomic studies of potato (Solanum tuberosum L.) under recurrent drought conditions identified potential memory genes, including genes involved in photosynthesis, carbohydrate metabolism, flavonoid metabolism, and others (Chen et al., 2019).

Given the observed associations of various important processes with plant stress memory, studying the effects of stress on the expression of genes of specific metabolic or signaling pathways can help identify marker genes. For example, analysis of the expression dynamics of AsCHS genes of the chalcone synthase family (flavonoid pathway) in garlic (Allium sativum L.) exposed to abiotic stressors identified only one out of eight genes as a potential biomarker (Anisimova et al., 2025). Another example: tracking changes in the expression of various PR genes in garlic cloves in response to priming with an elicitor (chitosan) and a biotic stimulus (infection with Fusarium proliferatum) identified candidate genes for markers of A. sativum memory of Fusarium infection (Filyushin et al., 2022).

Selected stress memory markers (both genes and metabolites) can be used to identify donors of a trait of the desired conditional (epigenetic) resistance to target stressors in crop plants (Aina et al., 2024). In plant genetic engineering, altering the expression of marker genes can facilitate the production of stress-resistant genotypes. For example, overexpression of individual genes from the WRKY family increases the resistance of tomato plants (Solanum lycopersicum L.) to phytopathogens (Bai et al., 2018), while overexpression of the DREB1A and OsPIL1 genes increases drought tolerance in A. thaliana (Kudo et al., 2017).

Tomato (S. lycopersicum) is an important vegetable crop, mainly grown in protected ground; soil salinity is considered one of the main factors reducing tomato crop yield (Guo et al., 2022). Epigenetic marks associated with the formation of salt stress memory in plants (Gallusci et al., 2023) and the mechanisms of salt tolerance in tomato are known (Guo et al., 2022). Among the genes, the expression of which is induced by salt stress, there are genes encoding transcription factors (TFs) of the DREB family (APETALA2/Ethylene Responsive Factor (AP2/ERF) superfamily), in particular the DREB2 (DEHYDRATION-RESPONSIVE ELEMENT-BINDING PROTEIN 2) subfamily (Bai et al., 2018; Guo et al., 2022). The tomato genome contains seven SlDREB2 genes (Maqsood et al., 2022).

The aim of this study was to evaluate SlDREB2 genes as possible marker genes for salt stress memory by profiling gene expression in two S. lycopersicum cultivars in response to the NaCl stimulus and during long-term post-stress recovery (memory phase).

## Materials and methods

The study involved plants of two salt-tolerant tomato (S. lycopersicum) varieties: the highly tolerant cv. Otradnyi and the moderately tolerant cv. Gnom, bred at the Federal Scientific Vegetable Center (FSVC, Moscow Region). Seeds were sown in the soil, and plants were grown until 6–8 leaves developed (experimental climate control facility, Federal Research Center for Biotechnology, Russian Academy of Sciences; day/night cycle – 16/8 h, 23/21 °C).

**Fig. 1.**
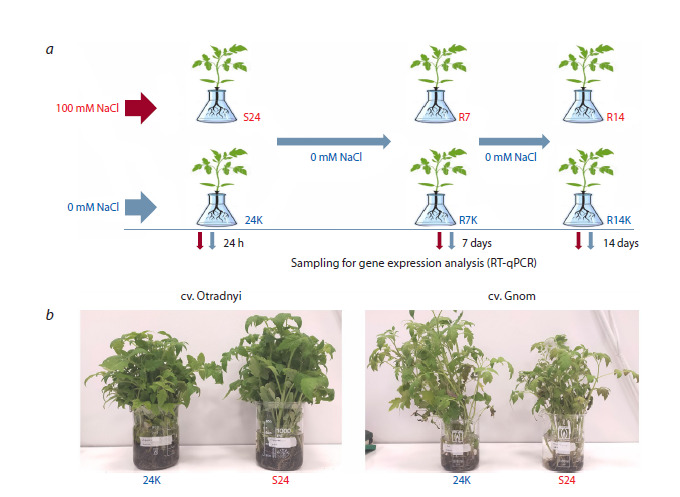
Experimental design: a – 24-h salt stress (100 mM NaCl (S24) and 0 mM NaCl (24K)) and post-stress recovery (7 (R7K, R7) and 14 (R14K, R14) days); b – photo of experimental (S24) and control (24K) plants of the Otradnyi and Gnom tomato varieties after 24 h of stress.

The obtained plants were exposed to salt stress. Namely, the plants (experimental and control) were transferred from soil to water (after shaking off and washing the roots) and after 1 h transferred to a liquid MS nutrient medium containing (experimental) and not containing (control) 100 mM NaCl. After 24 h, the experimental samples were returned to the MS medium without NaCl and kept for two weeks in parallel with the control. Leaf samples (all leaves from one plant; two biological replicates) were collected at the following time points: S24 (experimental, 24 h of stress exposure) and 24K (control); R7 and R7K (week of the post-stress period); R14 and R14K (two weeks of recovery) (Fig. 1a)

The collected samples were ground in liquid nitrogen and used for analysis of the expression of SlDREB2 subfamily genes using quantitative real-time PCR (RT-qPCR). Total RNA was isolated from 0.2–0.5 g of tissue (RNeasy Plant Mini Kit and RNase-free DNase set; QIAGEN, Germany) and used for cDNA synthesis (GoScript Reverse Transcription System; Promega, USA). The concentration of the preparation was determined (Qubit® Fluorometer, Thermo Fisher Scientific, USA; Qubit RNA HS Assay Kit, Invitrogen, USA), and 3 ng of cDNA was used for RT-qPCR with gene-specific primers (Table 1). Primers were designed based on available S. lycopersicum genome and transcriptome data (https:// www.solgenomics.net/; https://www.ncbi.nlm.nih.gov/). The reaction was carried out using the “2.5× Reaction Mixture for Real-Time PCR in the Presence of SYBR Green I and ROX” kit (Synthol LLC, Russia) on a CFX96 Real-Time PCR Detection System (Bio-Rad Laboratories, USA). The RT-qPCR program was as follows: 5 min at 95 °C, 40 cycles (15 s at 95 °C; 40 s at 60 °C). SlDREB2 gene expression was normalized to the reference genes Expressed and actin-7 (Efremov et al., 2020). The analysis was performed in two biological and three technical replicates. The obtained data were statistically processed and visualized using GraphPad Prism v. 9.5.1 (Two-Way ANOVA: multiple comparisons corrected with the Bonferroni test; GraphPad Software Inc., USA; https://www.graphpad.com/scientific-software/prism/). Excel was used to construct heatmaps and linear graphs based on expression data

**Table 1.**
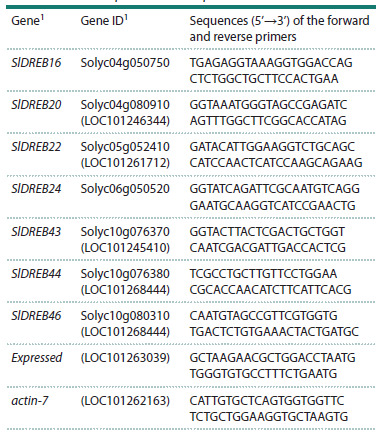
Primer sequences for RT-qPCR 1 The numbering and Solyc_IDs of genes are given according to (Maqsood et al., 2022); the corresponding NCBI_IDs of genes (if Solyc-protein homologs are present in the NCBI database) are given in brackets

## Results

In this study, we characterized the effects of salt stress and prolonged post-stress recovery on SlDREB2 gene expression in the leaves of tomato plants with high (cv. Otradnyi) and moderate (cv. Gnom) salt tolerance. After 24 h of NaCl exposure, as well as 7 and 14 days post-stress, plants of both varieties were visually indistinguishable from control, unstressed samples (Fig. 1b).

**Fig. 2.**
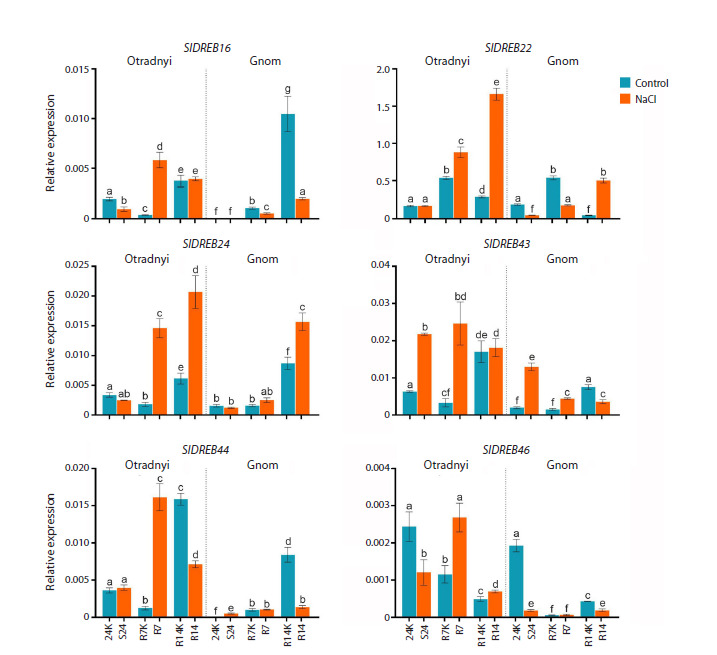
Expression pattern of SlDREB2 genes in the leaves of cv. Otradnyi and cv. Gnom tomato plants in response to salt stress for 24 h (24K and S24) and in the dynamics of post-stress recovery after 7 (R7K and R7) and 14 (R14K and R14) days. a–g Significant differences between expression levels within the variety (p &0.05).

Leaves from plants (control and experimental) at time points S24/24K, R7/R7K, and R14/R14K were collected and used to analyze the expression of SlDREB2 genes, the homologs of which in other plant species are known to be involved in the response to osmotic stress (Akbudak et al., 2018; Guo et al., 2022; Filyushin et al., 2023; Sun et al., 2025). Genes for analysis were identified based on the published characterization of the S. lycopersicum DREB gene family, in which the SlDREB2 subfamily is represented by seven intronless genes: SlDREB16, 20, 22, 24, 43, 44, and 46 (Maqsood et al., 2022). RT-qPCR analysis revealed that the SlDREB20 gene was not expressed in leaves in either the experimental or control plants, while the expression pattern of the remaining six genes was genotype-dependent (Fig. 2).

**Fig. 3.**
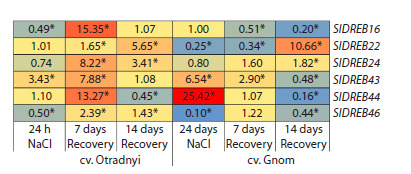
Heatmap of SlDREB2 gene expression in the leaves of cv. Otradnyi and cv. Gnom tomato plants in response to salt stress (24 h) and during post-stress recovery (7 and 14 days). Numerical data are presented as the ratio of values for experimental samples to the control (taken as 1). Significant differences in expression levels between the experiment and the control (p < 0.05).

The varieties differed in gene expression under control conditions, both in terms of the level at the 24K baseline (SlDREB16, 24, 43, and 44) and in the tendency to change over the measurement period (SlDREB16, 44, and 46). Only SlDREB22 showed a similar expression pattern between varieties under control conditions (Fig. 2). A heatmap was constructed based on the expression data (Fig. 3), clearly showing that in the case of the highly resistant cv. Otradnyi, only three genes (SlDREB22, 24, and 44) retained control expression levels after 24 h of stress. However, their transcript levels increased after one (~1.7, 8.2, and 2.4-fold) and two (~5.7, 3.4, and 1.4-fold) weeks of the recovery period. The expression of the remaining three genes decreased (SlDREB16, and 46) or increased (SlDREB43) after 24 h of stress and increased significantly at point R7. After two weeks of recovery, only two genes (SlDREB16, and 43) were expressed similarly to the control (Fig. 3).

In the leaves of the moderately resistant cv. Gnom, after 24 h of stress, gene expression increased (SlDREB43, and 44), decreased (SlDREB22, and 46), or remained unchanged (SlDREB16, and 24). After a week of recovery (R7), changes in expression were observed for three genes (SlDREB16, 22, and 43), whereas after two weeks (R14), all six genes were expressed differently from the control (Fig. 3).

Thus, a return to control expression was observed only long after the stress and only for SlDREB24, 44, and 46 (cv. Gnome, point R7), the expression of which at point R14 changed again (vs. control), as well as for the SlDREB16, and 43 (cv. Otradnyi, point R14) (Fig. 3).

**Fig. 4.**
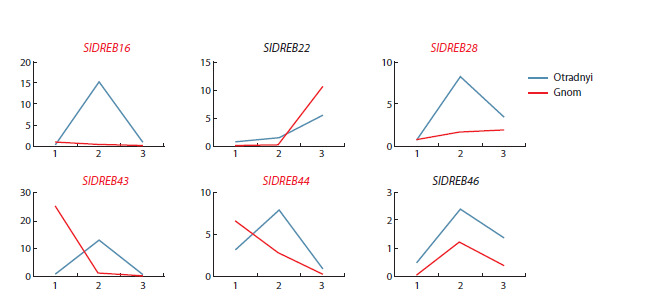
SlDREB2 gene expression patterns in the leaves of tomato cultivars Otradnyi and Gnom in response to salt stress (24 h) and during post-stress recovery (7 and 14 days), presented as linear graphs. Genes, the expression patterns of which show significant intervarietal differences in fluctuation trends, are highlighted in red.

To more clearly compare SlDREB2 expression patterns between cultivars, linear graphs were drawn using the expression data, expressed as the ratio of gene expression levels between the experimental and control conditions (Fig. 4). The graphs show that two genes (SlDREB22, and 46) have similar patterns of response to salt stress and memory phase in two analyzed cultivars. SlDREB16, 28, 43, and 44 genes showed different response patterns between varieties (Fig. 4).

To assess the possible dependence of the expression of the SlDREB16, 20, 22, 24, 43, 44, and 46 genes on the variability of their regulatory regions in tomato varieties, an in silico analysis of the promoters (1 kb) of these genes was performed in 10 tomato accessions (sequences were taken from the NCBI database). It was shown that the promoters of SlDREB16, 22, 24, 43, and 44 are highly conserved (0–2 polymorphisms (SNPs) per 1 kb), while the promoters of the SlDREB20 and 46 genes contain indels/SNPs (5/58 and 5/13, respectively).

## Discussion

In this study, we evaluated genes of the tomato SlDREB2 subfamily as potential marker genes for salt stress memory by profiling gene expression in two S. lycopersicum cultivars in response to NaCl and during the long-term post-stress recovery period (memory phase). The cultivars differed in their tolerance to salt stress (moderate in cv. Gnom and high in cv. Otradnyi). Cultivar tolerance can be regulated both by genetic variations governing gene expression in response to salt stress and by conditionally inherited epigenetic modifications, previously acquired as a result of salt priming and maintained by stress memory.

In the first case, genetic variations may be represented by genes and loci associated with the salt tolerance trait (Ismail, Horie, 2017). Differences in genes/loci may determine the degree of plant adaptability, as demonstrated by tomato genotypes carrying mutant TSS1 and TSS2 loci, which confer contrasting sensitivity to general osmotic stress and different mechanisms of salt tolerance (Borsani et al., 2001). Given the genetic regulation of NaCl tolerance, our experiment can be considered a primary stress for plants. In the second case, given the presumed presence of salt stress memory, the simulated salt stress conducted in this study will activate this memory. The third possible scenario involves genetic regulation of salt tolerance in one variety and epigenetic regulation in another.

Various transcriptome studies of NaCl exposure in plants suggest that key genes involved in salt stress memory are represented by TF genes of various families (Zhu et al., 2023), including the DREB family (Hassan et al., 2022). The importance of the latter is highlighted by the differential expression of DREB genes in response to salinity in wheat Triticum aestivum L. (Hassan et al., 2022), pepper Capsicum annuum L. (Sun et al., 2025), garlic A. sativum (Filyushin et al., 2023), and other species.

The choice of DREB2 subfamily genes from the two largest DREB subfamilies for analysis was determined by the fact that DREB1/CBF proteins play the greatest role in regulating cold tolerance (Shi et al., 2018), whereas DREB2 TFs are mainly involved in the response to osmotic stresses (Akbudak et al., 2018; Baillo et al., 2019). In the tomato genome, the DREB2 subfamily consists of seven genes: SlDREB16, 20, 22, 24, 43, 44, and 46 (Maqsood et al., 2022) (Table 1).

During the experiment, two tomato varieties were subjected to salt stress (24 h), followed by a long-term (14-day) memory phase (Fig. 1). Subsequent gene expression profiling (S24/24K–R7/R7K–R14/R14K) revealed significant genotype-specific variations in gene transcript levels in both control and stressed plants (Fig. 2), suggesting intervarietal differences in the mechanism of salt tolerance regulation

It was determined that during the long-term post-stress recovery period, gene expression values returned to control values either temporarily (SlDREB24, 44, and 46 in the Gnom variety at point R7; they changed again at point R14) or extremely slowly (SlDREB16 and 43 in the Otradnyi variety at point R14) (Fig. 3). This gene response in the case of both varieties corresponds to the feature of stress memory marker genes, the expression of which is maintained at an altered level for a long time during the recovery phase, while the expression of genes not associated with memory quickly returned to control values (Friedrich et al., 2019; Bäurle, Trindade, 2020; Jacques et al., 2021). This suggests that all six genes, SlDREB16, 22, 24, 43, 44, and 46, may function as salt stress memory marker genes in tomato plants.

Only two genes (SlDREB22 and 46) were shown to have a similar pattern of expression fluctuations between cultivars during the measurement period (S24/24K–R7/R7K–R14/ R14K) (Fig. 4). This suggests that the remaining four genes (SlDREB16, 28, 43, and 44) may play a role in determining differences in the mechanism of regulation of plant responses to salt stress between salt-tolerant genotypes of S. lycopersicum.

Overall, the performed assessment of the expression pattern of SlDREB2 subfamily genes in the leaves of two salttolerant tomato cultivars in response to NaCl and during the long-term memory phase suggests that these genes (except for SlDREB20) participate in the response of S. lycopersicum to excess salt in a genotype-specific manner. These genes may potentially serve as markers of stress memory linked to epigenetic regulation of plant adaptation to salt stress. The response of SlDREB2 genes to salt stress may also depend on genetic variations in the promoter regions of both the SlDREB2 subfamily genes themselves and the potential targets of the SlDREB2 TFs in S. lycopersicum accessions

The invariability in the regulatory sequences of the SlDREB16, 22, 24, 43, and 44 genes that we found (using in silico analysis of the promoters of the analyzed genes in 10 tomato cultivars/accessions) suggests that the conservation of these promoters may also extend to the cultivars used in this study. This suggests that the response of SlDREB16, 22, 24, 43, and 44 to salt stress is independent on intervarietal variations in their regulatory sequences. The SlDREB20 gene, the promoter of which is the most variable between accessions (58 SNPs), was not expressed in leaves; thus, the question of the dependence under consideration for this gene does not arise. At the same time, the expression level of SlDREB46 can be regulated by polymorphisms (13 SNPs), which requires additional studies of the SlDREB46 promoter in the tomato varieties used in the work, with a search for correlations between expression and the SNPs found.

The expression level of some DREB2 subfamily genes is positively associated with plant resistance to various abiotic stresses, as demonstrated by A. thaliana plants overexpressing the rice (Oryza sativa L.) OsDREB2B gene and exhibiting increased tolerance to drought and heat (Matsukura et al., 2010). It is suggested that in response to abiotic stress, the expression of DREB1/2 TFs is altered, which in turn regulate the transcription of target genes involved in plant defense. To date, data are available on 10 possible target genes of the DREB1/2 TF (A. thaliana) containing DRE/DRE-related cis-regulatory elements in their promoters, and six of these genes may be involved in the plant’s response to salt stress (Table 2) (Dubouzet et al., 2003; Matsukura et al., 2010).

**Table 2.**
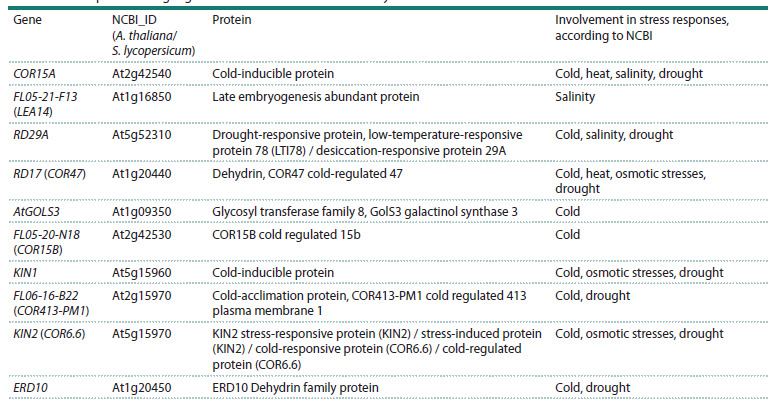
List of putative target genes of the A. thaliana DREB family TFs

## Conclusion

Thus, further studies of the structure and expression of SlDREB2 genes and their possible targets using repeated stress events interspersed with memory phases of varying duration, accompanied by expression analysis of genes presumably not linked to stress memory, are needed. The results of such studies can be used in breeding salt-tolerant tomato varieties

## Conflict of interest

The authors declare no conflict of interest.
